# Residual number processing in dyscalculia^☆^

**DOI:** 10.1016/j.nicl.2013.10.004

**Published:** 2013-10-14

**Authors:** Marinella Cappelletti, Cathy J. Price

**Affiliations:** a UCL Institute of Cognitive Neuroscience, London, UK; b Wellcome Trust Centre for Neuro-Imaging, UCL Institute of Neurology, London, UK

**Keywords:** Dyscalculia, Number cognition, Parietal lobe, Residual abilities, Superior and inferior frontal

## Abstract

Developmental dyscalculia – a congenital learning disability in understanding numerical concepts – is typically associated with parietal lobe abnormality. However, people with dyscalculia often retain some residual numerical abilities, reported in studies that otherwise focused on abnormalities in the dyscalculic brain. Here we took a different perspective by focusing on brain regions that support residual number processing in dyscalculia. All participants accurately performed semantic and categorical colour-decision tasks with numerical and non-numerical stimuli, with adults with dyscalculia performing slower than controls in the number semantic tasks only. Structural imaging showed less grey-matter volume in the right parietal cortex in people with dyscalculia relative to controls. Functional MRI showed that accurate number semantic judgements were maintained by parietal and inferior frontal activations that were common to adults with dyscalculia and controls, with higher activation for participants with dyscalculia than controls in the right superior frontal cortex and the left inferior frontal sulcus. Enhanced activation in these frontal areas was driven by people with dyscalculia who made faster rather than slower numerical decisions; however, activation could not be accounted for by response times per se, because it was greater for fast relative to slow dyscalculics but not greater for fast controls relative to slow dyscalculics. In conclusion, our results reveal two frontal brain regions that support efficient number processing in dyscalculia.

## Highlights

• Dyscalculics (DD) show congenital number impairment due to parietal abnormalities.
• However DD often show residual number skills which have not been studied before.
• We studied the brain networks supporting residual skills and individual differences
• DD: reduced parietal grey-matter, accurate but slower than controls in number tasks
• Faster DD responses over-activated two frontal areas

## 1 Introduction

Developmental dyscalculia (DD) is a congenital and specific learning disability affecting the understanding of numerical concepts and mathematical proficiency in the context of normal intelligence (American Psychiatric Association, 1994). People with dyscalculia – about 4–7% of the school-aged population (Shalev, 2007) – often make counting errors, have problems in performing arithmetical procedures, use developmentally immature and usually time-consuming problem solving strategies such as verbal or finger counting, and have difficulty in retrieving basic arithmetic facts from long-term memory (see Butterworth, 2005, 2010; Kaufmann et al., 2011; Rubinsten and Henik, 2009 for reviews).

Numerical impairments in dyscalculia have often been associated with functional and structural abnormalities that mainly involve the parietal lobes. For instance, in tasks that require the comparison or calculation of symbolic (larger number: 1 or 3?) or non-symbolic quantities (larger amount: ● or ●●●?), children and adults with dyscalculia have weaker activation compared to controls in and around the intra-parietal sulcus (IPS) and in some cases the inferior and pre-frontal areas (Kaufmann et al., 2009; Kucian et al., 2006; Molko et al., 2003; Mussolin et al., 2010a; Price et al., 2007; Rotzer et al., 2008). Other studies found that adolescent and adult with dyscalculia who were premature or had genetic disorders, had less grey-matter density in the left or right IPS (Isaacs et al., 2001; Molko et al., 2003). These results have been taken to suggest that the IPS is the most critical brain area for manipulating quantities (Butterworth, 2010; Wilson and Dehaene, 2007), or for connecting numerical symbols to their quantitative referent (Rousselle and Noël, 2007; Rubinsten and Henik, 2005, but see Mussolin et al., 2010b), although a more recent view highlights the multi-componential nature of dyscalculia (Fias et al., 2013).

A closer look at these neuroimaging studies indicates that differences between dyscalculics and controls are sometimes very specific. For instance, numerically-normal participants are slower to decide which of two numbers is larger when the numerical stimuli are close in magnitude (‘1’ vs ‘2’), than distant in magnitude (‘1’ vs ‘9’), and the numerical distance between the stimuli is inversely proportional to response times (Moyer and Landauer, 1967) and to parietal activation in controls (e.g. Pinel et al., 2001), but this effect is not observed in participants with dyscalculia (Kucian et al., 2006; Mussolin et al., 2010a; Price et al., 2007). Other differences in brain activation between people with dyscalculia and controls are often quite subtle or even non-existent (e.g. Kovas et al., 2008), and in some cases the associated statistics do not survive a standard correction for multiple comparisons (e.g. Kucian et al., 2006; Molko et al., 2003; Mussolin et al., 2010a). There may be many reasons why differences between adults with dyscalculia and controls are not always detected. One factor may be related to the type of control tasks used in fMRI experiments, which is usually subtracted from the experimental task(s) of interest. Some control tasks involved implicit quantity processing, for example when participants were required to compare shades of colours or of grey (Kucian et al., 2006; Mussolin et al., 2010a). When subtracting these quantity-based control tasks from the experimental numerical tasks, potential differences between the tasks and between adults with dyscalculia and controls may have been cancelled out because the experimental and the control tasks are both based on quantity processing.

Since most of the current imaging studies on dyscalculia report the parietal lobes among the main areas of interest, a second factor concerns the role of these areas in response-selection and comparison processes. It is well known that parietal areas are engaged by these processes as well as numerical processes (e.g. respectively Corbetta and Shulman, 2002; Cohen Kadosh et al., 2008; Dehaene et al., 2003). We have previously been able to tease apart parietal areas related to numerical processes from those related to response-selection processes, which typically correlate with reaction times (Cappelletti et al., 2010). By factoring out response times, we identified right parietal areas that were selective to semantic processing of numbers more than words from left parietal areas linked to response-selection processes that numbers share with other non-number semantic categories. These results therefore highlight the different roles of the left and right parietal regions when processing numbers, and the importance of factoring out response-related factors when characterizing parietal activations in dyscalculia.

A third factor that may account for previous inconsistent results or undetected differences between people with dyscalculia and controls is related to individual differences in the dyscalculic sample. Dyscalculia is known to be heterogeneous (Rubinsten and Henik, 2009), although previous studies have only focused on group effects. However, these effects may potentially hide differences across individual dyscalculics. One reason why it is important to consider individual differences in dyscalculia comes from the observation that people with dyscalculia often retain some residual abilities to perform numerical and quantity tasks especially when their responses are untimed. For instance, participants with dyscalculia have been reported to be accurate at comparing the value, height or greyness of numerical stimuli, at naming numbers and at comparing the duration of stimuli (e.g. Cappelletti et al., 2011; Censabella and Noël, 2005; Kaufmann et al., 2009; Kucian et al., 2006; Landerl et al., 2004; Rotzer et al., 2008; Rubinsten and Henik, 2005). This suggests that dyscalculics may have developed strategies to overcome their quantity and number impairment. Yet, the focus of research in dyscalculia has so far been in terms of their impaired number skills observed at a group level rather than residual numerical abilities which may vary from one individual to another.

### 1.1 This study

To investigate the impaired brain systems in dyscalculia as well as those supporting residual number processing (defined as number accuracy not differing from controls), several methodological novelties were introduced in this study: first we used identical semantic and baseline tasks for numbers and for another non-numerical category, i.e. written object names; this allowed us to distinguish between any effect related to general semantic manipulations (such as extracting meaning from numbers symbols or names) and specific to number (like quantity manipulation). Secondly, we used a baseline control task that did not require any quantity processing but instead was a categorical colour-decision task on the identical numerical and object-name stimuli. Third, we controlled for RT-related effects similar to our previous study of numerically-normal participants (Cappelletti et al., 2010); this allowed us to identify any number-parietal activation that was not contaminated by RT effects. Fourth, as well as looking at group effects, we also examined individual differences in behavioural and neuronal profiles. Finally, we focused on adult participants with dyscalculia rather than children who are more commonly investigated (e.g. see Kaufmann et al., 2011 for a review). This allowed us to tease apart activations that may reflect developmental changes (e.g. Ansari and Dhital, 2006) from activations that more uniquely reflect number processing. For instance, frontal activations in children sometimes reflect greater reliance on attentional and working memory resources compensating for undeveloped parietal regions, which in adults support automatic processing of numbers (Ansari et al., 2005; Rivera et al., 2005).

More broadly, looking at residual numerical performance in dyscalculia may offer a perspective into brain plasticity, i.e. how the human brain is capable of adapting to cope with the need or pressure to use numbers despite the difficulties in doing so.

## 2 Material and methods

### 2.1 Participants

Overall one hundred and twelve right-handed, MRI compatible, native English speakers with normal or corrected to normal vision gave informed written consent to participate in the study. Eleven of these participants had developmental dyscalculia, the remaining one hundred and one were numerically-normal controls. The study was approved by the National Hospital and Institute of Neurology's joint ethics committee.

#### 2.1.1 Control subjects

There were three groups of numerically-normal participants. The first group served as control for the fMRI study and one of the MRI analyses; it included twenty-two right-handed numerically-normal participants comprising twelve females with a mean age of 54.55 years. Their wide age range (22–74) provided a normal source of inter-subject variability from which to assess variability in the participants with dyscalculia. We have described the fMRI results from these control subjects in an earlier publication (Cappelletti et al., 2010). Here we used the same sample to assess normal and abnormal activation in our population of dyscalculic participants. We also used this control sample to test whether any abnormal activation corresponded to structural abnormalities using voxel-based morphometry (VBM, Ashburner and Friston, 2000).

The second control group of numerically-normal participants was recruited to increase the power of the VBM analysis when assessing structural abnormalities in dyscalculia. This group included 29 right-handed healthy females with a mean age of 42.12 years (range 24–70). The third group of numerically-normal participants comprised 50 new subjects (33 females, mean age = 35.6, range 19–76) that took part in previous studies (Cappelletti et al., under review-a,b) and that were compared to dyscalculics only in some of the out-of-scanner behavioural assessments, specifically number comparison and numerosity discrimination.

#### 2.1.2 Adults with developmental dyscalculia

Eleven adults with dyscalculia were studied (all females; mean age: 42.82 years, range 25–70). Dyscalculia was diagnosed before participants were invited to take part in the study. The diagnosis was based on the Dyscalculia Screener (Butterworth, 2003), and corroborated by performance in: (i) a standardised mathematical task, i.e. the Graded Difficulty Arithmetic test (GDA, Jackson and Warrington, 1986); (ii) the arithmetic subtest of WAIS-R (Wechsler, 1986); (iii) a number comparison task; and (iv) a task consisting of discriminating the numerosity of clouds of dots, which allows the calculation of the Weber fraction (Halberda et al., 2008), an index of accuracy sensitive to dyscalculia (Mazzocco et al., 2011; Piazza et al., 2010). General intelligence was also assessed with the WAIS-R (Wechsler, 1986). See Table 2 for the tests used and Appendix A for further details.

To be classified as dyscalculic, a participant had to obtain: (i) a standardised score below 81 on at least one of the two tasks of the capacity subscale of the Dyscalculia Screener (see below; test average of the nationally standardised score = 100, SD = 15); (ii) a score below 2 standard deviations of either the controls mean performance or the 50th percentile in the number comparison, the numerosity discrimination and the standardised arithmetic tasks; and (iii) an IQ score within the normal range (full-scale IQ not below 80).

Dyscalculics' IQ was average or high average, suggesting preserved intellectual functioning (see Table 1). In contrast, all 11 participants with dyscalculia obtained a standardised score below the cut-off point in the Dyscalculia Screener; they were also impaired in the two standardised calculation tests. In the number comparison task, adults with dyscalculia showed an abnormally large distance effect such that the time required to discriminate between stimuli numerically close was abnormally long relative to participants in Control Group 3 [t(59) = 8.2, *p* < 0.02] and consistent with some previous studies (e.g. Ashkenazi et al., 2009; Holloway and Ansari, 2008; Mussolin et al., 2010a). In the numerosity discrimination task, participants with dyscalculia showed an abnormally large Weber Fraction relative to numerically-normal participants [Control Group 3, t(59) = 4.5, *p* < 0.001], such that in dyscalculics the numerosity in the two sets of dots had to be significantly further apart to be correctly discriminated relative to numerically-normal participants (Control Group 3). This pattern of performance in adults with dyscalculia resembles that of dyscalculic children (e.g. Mazzocco et al., 2011; Piazza et al., 2010). Group differences in the number comparison and the numerosity discrimination tasks were maintained even when dyscalculics were compared to gender-matched participants only [*N* = 33 of Control Group 3, t(42) = 6.3, *p* < 0.03 and t(42) = 4.9, *p* < 0.001 respectively].

### 2.2 fMRI experimental design

The experimental design was identical to that previously used to investigate the number system in numerically-normal participants and in neurological patients (Cappelletti et al., 2010, 2012). It independently manipulated stimulus type (numerals, e.g. ‘23.07’ or object names, e.g. ‘desk’) and task. In all conditions, participants were simultaneously presented with two stimuli (either numbers or written object names), and a two-word question. One word referred to the type of information that needed to be attended to (see Section 2.2.2) and the other word indicated the type of stimulus (number or object).

**Fig. 1:**
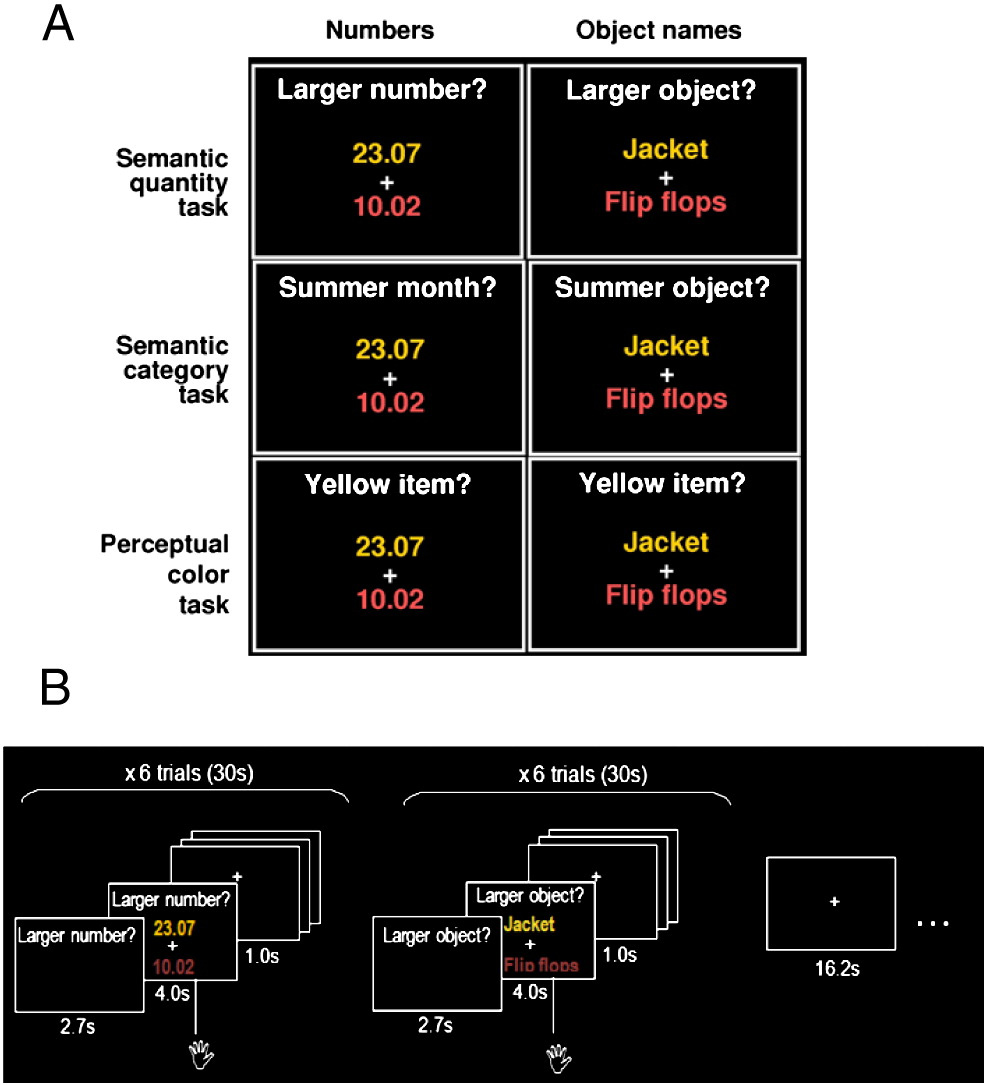
Experimental design. (A) The same semantic quantity and category tasks and perceptual colour decision tasks were used with pairs of Arabic numbers (left panel) and object names (right panel) which were presented in one of four possible colours red, yellow, blue, green. For each semantic task, one of two possible questions was presented in different blocks in counterbalanced order i.e. larger/smaller, more/less, summer/winter, working/sleeping. In each trial (B), participants viewed pairs of stimuli presented one above the other with a fixation cross in the middle of the computer screen. Subjects were instructed to indicate with a button press which of the two stimuli was the correct response to a question consisting of two keywords presented above the upper stimulus before and during the stimulus display. The 6 different conditions (3 tasks × 2 stimuli) were blocked (6 trials per block) and fully counterbalanced between and within subjects. In each task, the first block consisted of 6 trials with numerical stimuli or object names, followed by another 6-trial block of the same task with object names or numerical stimuli in a counterbalanced order. Presentation of blocks of the same task with both stimuli was followed by about 16-second rest period where subjects were asked to maintain fixation on a cross in the middle of the computer screen. Trials where the correct answer was the upper or the lower stimulus were presented in equal proportion, see Cappelletti et al., 2010 for more details.

The tasks were categorized on 2 levels. The first involved semantic decisions on number or object names (Fig. 1A, top and middle row), the second was a perceptual colour decision task that involved selecting one of two stimuli on the basis of their colour (Fig. 1A, bottom row). The semantic task consisted of 2 subtasks: (i) a quantity task, which required a finger-press response to indicate the larger (or more numerous) or smaller (or less numerous) of two numbers or two objects; and (ii) a category task, which required a finger-press response to indicate which of two numbers or objects was associated with a summer or winter date or sleeping/working hour see Fig. 1.

#### 2.2.1 Experimental stimuli

A total of 144 Arabic numbers and 144 object names were used. Arabic numbers were presented as pairs of digits, each separated by a dot, e.g. 23.07. They referred to a linear dimension of quantity, to dates (e.g. 23rd July) or to times (e.g. seven minutes past eleven at night). Object name stimuli referred to concrete, countable objects whose size could be unambiguously identified and that could be used in both the quantity (e.g. larger object: ‘sailing boat’ or ‘desk’?) and non-quantity tasks (e.g. working object: ‘sailing boat’ or ‘desk’?). In the scanner, the two stimuli were presented one above and one below a central fixation point (see Fig. 1B). Each stimulus appeared three times, one for each of the quantity, categorical and colour decision tasks. For more details about the stimuli used see Cappelletti et al. (2010).

#### 2.2.2 Task instructions

Participants were told that they would see pairs of numbers or object names and that the instructions would be presented above the top stimulus in the form of a two-word question. On every trial, participants were instructed to make a key press response to indicate which stimulus corresponded to the correct answer to the question. They were asked to press the upper key of a two-button keypad to select the upper stimulus and the lower key to select the lower stimulus. Trials where the correct answer was the upper or the lower stimulus were presented in equal proportion.

For the number semantic tasks, participants were also told that the number stimuli could indicate either: (1) quantities, (2) dates, or (3) times. They were presented with four different questions for each type of task. For the quantity task, the questions were: (i) larger number? (ii) smaller number? (iii) more numbers? (vi) less numbers? For the category task, the four questions were: (i) summer month? (ii) winter month? (iii) working time? (vi) sleeping time? For the larger/smaller and more/less questions, participants were told that numbers referred to an amount and that they should choose the larger (or smaller) number in each pair irrespective of the wording of the question (i.e. “larger” or “more” and “smaller” or “less”). For summer/winter questions, participants were told that each number indicated either a summer or a winter month in the Northern hemisphere. They were told that summer months were ‘June’, ‘July’, and ‘August’ and winter months were ‘December’, ‘January’ and ‘February’ and that these months followed a day (1–31) separated with a dot (13.07) rather than the more familiar slash (13/07). Participants were instructed to select either the summer or the winter month in each pair of stimuli depending on the question. For the working/sleeping questions, participants were told that working or sleeping times were in terms of a 24-hour clock; and that working times were between 8am and 6pm, and sleeping times were between 10pm and 7am. Participants were told not to consider jobs that include night shifts.

In the perceptual colour decision task, participants selected the stimulus whose font was in one of 4 pre-defined possible colours (yellow, green, red, and blue).

For object names, the instructions were the same as those for the numbers; therefore for the categorical task participants were required to identify the working/sleeping item among two (e.g. ‘bed vs desk’) or the summer/winter object (e.g. ‘bikini vs boots’). For the quantity task, participants were instructed to select the larger/smaller object among two (e.g. ‘bed vs chair’) or the more/less numerous object (e.g. ‘stars vs moon’ or ‘snowflakes vs snowman’). Note that the latter questions (‘larger/smaller’ and ‘more/less’) differed in the tasks with object names and with number where ‘larger’ was equivalent to ‘more’ and ‘smaller’ to ‘less’. Prior to the fMRI experiment, participants underwent a practice session in order to familiarize themselves with the task procedure.

#### 2.2.3 Presentation parameters

The 6 different conditions (quantity, category and perceptual-decision tasks × 2 stimuli) were blocked and fully counterbalanced between and within subjects. We used two versions of the same experiments (paradigm 1, P1 and paradigm 2, P2 with 8 and 14 participants respectively). These paradigms differed in terms of the hand used to respond and timing parameters (see below and Table 1 in Cappelletti et al., 2010). However, because these factors were consistent across conditions, none influenced the range of effects observed in within-subject comparisons of semantic relative to colour judgements (Cappelletti et al., 2010).

Participants with dyscalculia were scanned with the timing parameters of paradigm 2. Specifically, in P2 tasks with numbers and object names were each presented in 36 blocks with six stimuli per block, 24 blocks of the semantic tasks (12 quantity and 12 categorical tasks), and 12 of the colour decision task. The 72 blocks were divided in 4 sessions (ABCD) in which 12 blocks of semantic tasks and 6 blocks of perceptual colour decision tasks were presented in pseudo-random order in each session, counterbalanced with an equal proportion of written word stimuli. There were four possible sequences of the sessions (ABCD, CADB, BDAC, DCBA), with participants randomly assigned to each sequence. Each block began with a question that appeared before the first trial for 2.7 s and remained on the screen for the duration of the block. Each pair of stimuli remained on the screen for four seconds, and was followed by an inter-stimulus interval of one second before the next pair appeared. A fixation cross lasting 16.2 s was then presented between blocks (see Fig. 1B).

#### 2.2.4 Data acquisition

A Siemens 1.5T Sonata MRI scanner (Siemens Medical, Erlangen, Germany) was used to acquire both anatomical and functional images.

##### 2.2.4.1 Functional imaging data

Functional T2*-weighted echoplanar images with BOLD contrast comprised 40 (P1) or 30 (P2) oblique axial slices of 2 mm thickness with 1 mm slice interval and 3 × 3 mm in-plane resolution. 216 (P1) or 260 scans (P2) were acquired in three (P1) or four (P2) sessions. Effective repetition time (TR) was 3780 ms (P1) and 2700 ms (P2) with TR and Stimulus Onset Asynchrony not matching to allow for distributed sampling of slice acquisition across the experiment (Veltman et al., 2002). Because acquisition factors were consistent across conditions, none influenced the range of effects from within subject comparisons of semantic relative to colour judgements. To avoid Nyquist ghost artefacts a generalized reconstruction algorithm was used for data processing.

##### 2.2.4.2 Structural imaging data

High-resolution anatomical images were acquired using a T1-weighted 3-D Modified Driven Equilibrium Fourier Transform (MDEFT) sequence (TR = 12.24 ms; TE = 3.56 ms; field of view = 256 × 256 mm; voxel size = 1 × 1 × 1 mm). The images were spatially normalised to Montreal Neurological Institute (MNI) space and segmented into grey and white matter using the unified segmentation algorithm (Ashburner and Friston, 2005). Subsequently, a Diffeomorphic Anatomical Registration Through Exponentiated Lie Algebra (DARTEL) was performed for inter-subject registration of the grey-matter images. To ensure that the total grey-matter volume was retained before and after spatial transformation, the image intensity was modulated by the Jacobian determinants of the deformation fields. The registered images were then smoothed with a Gaussian kernel (FWHM = 8 mm) and were then affine transformed to MNI stereotactic space using affine and non-linear spatial normalisation, for multiple regression analysis.

## 3 Data analysis and results

Since our sample of adults with dyscalculia was composed of females only, and because there is evidence of gender effects in numerical processing (e.g. Knops et al., 2006), we report behavioural and neuroimaging effects obtained by comparing our dyscalculic sample to gender-matched participants only, as well as the full sample of gender mixed participants.

### 3.1 Out-of-scanner behaviour

Behavioural data were analysed using parametric tests (ANOVA and t-tests) with significance set at a *p* value of 0.05. Additional analyses of individual dyscalculic data were performed following the Crawford and colleagues' approach (Crawford and Garthwaite, 2002; Crawford et al., 1998); specifically, we used a two-tailed significance test to compare each participant with dyscalculia with the numerically-normal participants. This test treats each individual participant as a sample, affording the comparison of a single individual with the control group (Crawford and Garthwaite, 2002; Crawford et al., 1998).

### 3.2 In-scanner behaviour

#### 3.2.1 Accuracy

**Fig. 2:**
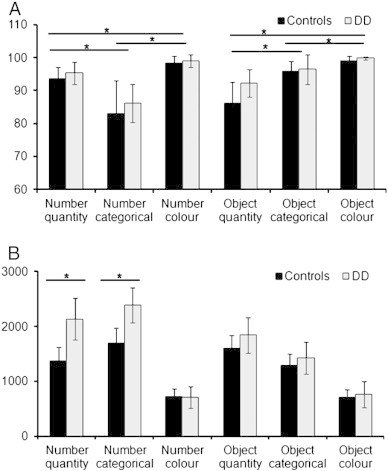
In scanner accuracy, percent correct and response times (RTs) with standard error (SE) in participants with dyscalculia and in control participants. (A) Adults with dyscalculia performed accurately on all tasks, showing no significant difference with controls. However, (B) participants with dyscalculia were significantly slower than controls only in number semantic tasks (both number quantity and number categorical).

An analysis of variance (ANOVA) on the mean accuracy with stimulus (numbers and object names) and task (quantity, categorical, colour decision) as within-subject variables and group (dyscalculics and Control Group 1) as between-subject variable found no effect of group (dyscalculics versus controls) and no group by task or group by stimulus interaction. Across subjects, there was a main effect of task [F(2,62) = 54.1, *p* < 0.001], and of stimulus [F(1,31) = 23.1, *p* < 0.001]. The stimulus effect arose because of higher accuracy for words than numbers [92.6 vs 89.5%, sd = 4.3 vs 3.9, t(32) = 4.2, *p* < 0.001], the task effect arose because of higher accuracy for colour than quantity or categorical judgements [95 vs 81.23 and 77.8%, sd = 1.3, 4.2 and 5.1 respectively, t(32) = 10.4, *p* < 0.001 and t(32) = 10.1, *p* < 0.001]. The 2-way interaction between task and stimuli was also significant [F(2,62) = 54.2; *p* < 0.001; see Fig. 2A], because accuracy was higher in the number relative to the word quantity judgements [94.7 vs 87.8%, sd = 4.1 vs 6.7, t(32) = 5.5, *p* < 0.001] and the word relative to the number categorical judgements [96.2 vs 83.4%, sd = 3.6 vs 8.4, t(32) = 9.3, *p* < 0.001].

Additional analyses with female control participants only (Control Group 1, *N* = 12) and with the same within-subject variables as the previous analyses, showed equivalent results. In particular, there was no effect of group and no group by task or group by stimulus interactions.

#### 3.2.2 Response times

The identical analyses were conducted on mean response latencies of correct answers.

Again there was no main effect of group [Control Group 1, F(1,31) = 8.6, *p =* 0.36, *ns*], but there was a significant 3-way interaction between group, task and stimulus [F(2,62) = 21.1, *p* < 0.001] because participants with dyscalculia were slower than controls in the number tasks (1744 ms vs 1401 ms; sd = 394 ms vs 217 ms, F(2,62) = 32.7, *p* < 0.001) but not in the equivalent tasks with object names (1342 ms vs 1298 ms, sd = 259 ms vs 173 ms, F(2,62) = 3.6, *p* = 0.16, *ns*). Further independent sample *t*-test corrected for multiple comparisons showed a significant group difference in the number quantity task (e.g. larger number: ‘23.07’ or ‘10.02’?; mean RTs: Dyscalculics = 2133 ms, sd = 580 ms; controls = 1391 ms, sd = 224 ms, t(31) = 5.3, *p* < 0.001) and the number categorical conditions only (e.g. summer month: ‘23.07’ or ‘10.02’?; Dyscalculics = 2390 ms, sd = 519 ms; controls = 1654 ms, sd = 318 ms, t(31) = 5.1, *p* < 0.001) but not in the colour decision task with numbers [Dyscalculics = 710 ms, sd = 195mms; controls = 726 ms, sd = 147 ms, t(32) = 0.2, *p* < 0.8, *ns*, see Fig. 2B).

Across subjects, there was a significant main effect of task [F(2,62) = 476.2; *p* < 0.001] because responses were faster for colour than quantity or categorical tasks [729 ms vs 1638 ms and 1613 ms respectively, sd = 166 ms vs 376 ms and 362 ms, t(32) = 15.7, *p* < 0.001 and t(32) = 16.2, *p* < 0.001]. There was also a significant main effect of stimulus [F(1,31) = 52.6; *p* < 0.001] because response times were faster for words than numbers [1317 ms vs 1523 ms, sd = 197 ms vs 319 ms, t(32) = 5.1, *p* < 0.001]. A significant 2-way interaction between task and stimulus [F(2,62) = 107.9; *p* < 0.001] arose because response times were faster in the word than number categorical judgements [1372 ms vs 1900 ms, sd = 253 ms vs 524 ms, t(32) = 8.4, *p* < 0.001].

Additional analyses with female controls only (Control Group 1, *N* = 12) showed equivalent results with a main effect of task [F(2,42) = 298.7, *p* < 0.001], of stimulus [F(1,21) = 34.3, *p* < 0.001], a significant 2-way interaction between task and stimuli [F(2,42) = 70.1; *p* < 0.001], and a significant 3-way interaction between task, stimulus and group [F(2,42) = 12.3, *p* < 0.001].

A median-split (e.g. Fukuda and Vogel, 2011; Tseng et al., 2012) was used to divide participants with dyscalculia in slow and fast based on performance in the in-scanner number semantic tasks (i.e. quantity and categorical judgements). We chose these tasks as they were the only ones among those run in the scanner in which dyscalculics and controls (Control Group 1) differed. This procedure divided participants with dyscalculia in high performers (*n* = 6; mean RTs = 1918 msec) and low performers (*n* = 5; mean RTs = 2673 msec). High and low dyscalculic performers significantly differed in speed [t(9) = 3.1, *p* = 0.01] but not accuracy [t(9) = 0.2, *p* = 0.8, *ns*] in the number semantic tasks. Low performing dyscalculics differed significantly from the numerically-normal participants in Control Group 1 [t(25) = 8.3, *p* < 0.001], whereas high performers did not [t(26) = 1.6, *p* = 0.1, *ns*]. Our fMRI and MRI analyses examined whether these behavioural differences may be reflected in differences in the brain correlates of dyscalculia.

### 3.3 Functional imaging data

Functional and structural image analyses were performed using Statistical Parametric Mapping software (SPM8 software, Wellcome Trust Centre for Neuroimaging, London; http://www.fil.ion.ucl.ac.uk/spm) running under Matlab 7.3 (MathWorks, Sherbon, MA, USA).

The first four (P1) and six (P2) volumes of each fMRI session were discarded and the remaining 212 (P1) and 254 (P2) volumes for each session were used for the analysis. Scans were realigned, unwarped and spatially normalised (Friston et al., 1995) to the Montreal Neurological Institute (MNI) standard space. Functional images were then smoothed in the spatial domain with a Gaussian kernel of 6 mm FWHM to improve the signal to noise ratio. A high pass filter was used with a cut-off period of 128 s.

#### 3.3.1 First level analysis

In a first level analysis activation for correct responses for each condition was compared to fixation according to the general linear model (Friston et al., 1995). Specifically, the functional data were modelled in an event related fashion, i.e. responses to individual stimuli were modelled within each block (e.g. Mechelli et al., 2003; Price et al., 1999) with six categorical regressors (1 or 0) corresponding to the correct responses to each of the conditions (three tasks: quantity, category, i.e. semantic, and perceptual colour decision x two stimuli: numbers and object names) and an extra regressor modelling all incorrect responses.

From the first level analysis of each participant, we computed 2 contrast images: number semantics (category and quantity) compared to the colour decision on numbers; and word semantics (category and quantity) compared to the colour decision on words. As shown previously (Cappelletti et al., 2010), parietal activation increases for the numerical tasks that explicitly require semantic processing compared to the colour decision on numbers, even though numerical information is sometimes reported to be automatically activated in the absence of an explicit task (e.g. Dehaene et al., 2003; Klein et al., 2011).

#### 3.3.2 Second level analysis

Our aim was to identify differences between people with dyscalculia and controls, particularly those that were number selective. This required a second level analysis of variance (ANOVA) with two factors (3 groups and 2 contrasts) and a total of 6 conditions. The three participant groups were adults with dyscalculia, P1 controls and P2 controls. The 2 contrast images, for each participant, were those computed at the first level (i.e. number semantics relative to colour or word semantics relative to colour).

In addition, for each subject and each condition, age and mean response times of correct answers only were entered as 2 continuous covariates. Response times were included to remove between-subject variability that was related to response times. This was to minimize inter-subject variability that was proportional to response times. From this analysis, we identified brain areas where activation for number semantics was greater than in the colour baseline task: (1) in both dyscalculics and control subjects (Control Group 1), (2) in control subjects more than in dyscalculics, and (3) in dyscalculics more than control subjects. To ensure that group differences were the result of semantic activation relative to colour decision rather than deactivation relative to colour decision, we inclusively masked with the number semantic contrast for both controls and dyscalculics (contrast 1), for controls only (contrast 2) and for dyscalculics only (contrast 3).

The statistical threshold was set at *p* < 0.05 after family wise error correction for multiple comparisons (height or extent) for contrasts 1–3, and at *p* < 0.001 uncorrected for all inclusive masks.

### 3.4 Functional imaging results

#### 3.4.1 Main effect of semantics, common to adults with dyscalculia and controls

Across control and dyscalculics groups, semantic activation for numbers relative to colour baseline increased in the bilateral intra-parietal sulcus (IPS), the right supra-marginal gyrus (SMG), and the right inferior frontal cortex (see Table 3A). Activations in these commonly activated areas did not differ in the two groups, irrespective of whether the control group included all participants (all *p* > 0.35), or females only (*p* > 0.1).

#### 3.4.2 Differences between adults with dyscalculia and controls

**Fig. 3:**
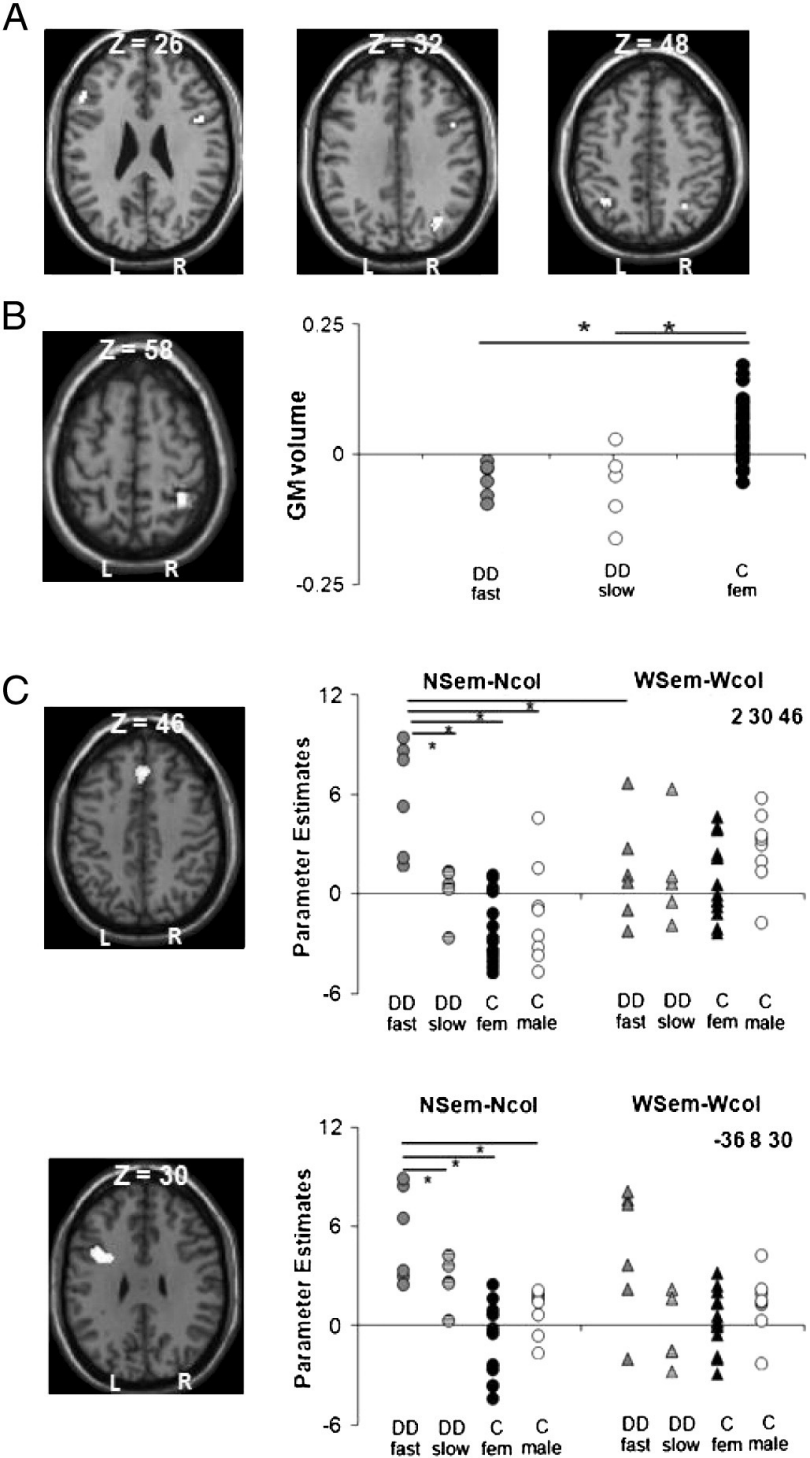
Neuronal profile in participants with dyscalculia. Brain regions displayed on axial sections of a template brain showing (A) common activations in participants with dyscalculia and 22 numerically-normal participants in right inferior frontal and bilateral IPS; (B) structural abnormalities: significant reduction in IPS grey-matter volume in 11 females with dyscalculia relative to 29 female controls. Right panel plots an index of IPS grey-matter volume reduction in participants with dyscalculia relative to controls; (C) functional abnormalities: over-activation in the right superior and left inferior frontal regions in participants with dyscalculia relative to 22 controls in number and word semantic relative to number and word colour judgements. Right panels: plot of the parameter estimates showing larger effects in fast relative to slow dyscalculic performers and controls in both frontal areas. Asterisks indicate significant differences.

At a corrected level of significance, there were no areas in which participants with dyscalculia showed less activation than controls in either the number or the word semantic tasks. In contrast, during number semantic tasks greater activation in adults with dyscalculia than controls was observed in the right superior frontal gyrus (in close proximity of the pre-SMA), and in the left inferior frontal gyrus/sulcus, which survived correction for multiple comparisons across the whole brain. There was a group-by-condition interaction in the superior frontal gyrus indicating that this effect was significantly greater for number semantics than word semantics in the dyscalculic group. The results did not change when the control group was limited to females only (i.e. gender matched to the dyscalculics) (see Table 3B and Fig. 3).

#### 3.4.3 Correlation between behaviour and brain activation

First we examined whether and how activation in the two regions that were more activated in adults with dyscalculia than in controls during number semantic tasks was related to in-scanner RTs. In dyscalculics, we found a negative correlation which indicated that activation in both regions increased as response times decreased [*r* = − 0.88; F(1,10) = 29.5 *p* < 0.001 and *r* = − 0.80; F(1,10) = 16.6, *p* < 0.001 in superior frontal and inferior frontal regions respectively]. Thus activation was higher for faster dyscalculics than slower dyscalculics [t(9) = 3.0 and 2.1; *p* = 0.01; and 0.06 in superior frontal and inferior frontal regions respectively]. The same two regions were not activated in controls who were as fast as fast dyscalculics, suggesting that activation was not therefore related to responses times per se.

Second, we tested whether activation in the two frontal regions that were more activated in adults with dyscalculia than controls (Control Group 1) during number semantic tasks was related to out-of-scanner behavioural measures. We found that activation increased with faster RTs in the number comparison task [*r* = − 0.61, F(1,10) = 5.5, *p* < 0.04; and *r* = − 0.5, F(1,10) = 4.3, *p* < 0.05 superior frontal and inferior frontal regions respectively], but not in other number tasks (e.g. addition and multiplication problems, *p* > 0.1) that also varied in speed of response, but had similar demands on stimulus and response-selection procedures.

Third, we tested whether performance in the in-scanner number tasks correlated with performance in brain regions other than the right superior and left inferior frontal areas. We focused specifically on the right posterior IPS (x = 32, y = − 72, z = 32) as this brain region was more activated for our number semantic tasks relative to object name semantic tasks (see Table 3). We found a significant correlation between dyscalculics' right posterior IPS activation and performance in the in-scanner number semantic tasks [*r* = − 0.63; F(1,10) = 5.8, *p* = 0.001], with a significant difference between fast and slow dyscalculics performers in the strength of the correlation between IPS activation and in-scanner performance [*r* = 0.78 and *r* = 0.08 respectively, Fisher transformation, *Z* = 2.93, *p* = 0.003].

### 3.5 Structural imaging data

Voxel-based-morphometry (VBM) analyses of structural MRI images were performed on the spatially normalised grey-matter (GM) images (see Section 2.2.4.2) using the grey-matter volume in each voxel as the dependent variable whilst controlling for age, gender and intracranial volume. Global signal intensity differences were removed using proportional scaling. We used cluster-level statistics with a non-stationarity correction, which is essential to adjust cluster sizes according to local ‘roughness’ (Hayasaka et al., 2004).

We found no brain areas where grey matter volume was significantly different in participants with dyscalculia compared to 29 gender-matched controls in a whole brain search (*p* > 0.05, after family-wise error correction for multiple comparisons). We then restricted our search volume to a spherical right parietal area (5 mm radius) centred on the co-ordinates x = 34 y = − 50 z = 52. These co-ordinates correspond or are in close proximity to those previously reported as under-activated in DD relative to controls (Kaufmann et al., 2011: x = 34 y = − 50 z = 52; Mussolin et al., 2010a: x = 41y = − 45 z = 56; Price et al., 2007: x = 30 y = − 50 z = 52). In this pre-selected right parietal ROI, participants with dyscalculia showed significantly reduced grey-matter volume relative to 29 gender-matched controls [t(38) = 5.4, *p* < 0.001, see Fig. 3]. Moreover, nine out of the eleven participants with dyscalculia had significantly reduced grey-matter volume relative to controls (see Table 4) according to the Crawford and colleagues' approach (Crawford and Garthwaite, 2002; Crawford et al., 1998). This involved measuring how deviant each participant with dyscalculia was from controls in terms of grey-matter volume in this region. We extracted the grey-matter signal for all participants and normalised the dyscalculics' values based on the controls' average and standard deviation. Abnormalities in dyscalculics' brain structure corresponded to values below 1 (borderline) or 2 (defective) standard deviations from the 29 gender matched controls.

Finally, we investigated whether parietal grey matter volume in adults with dyscalculia was related to activation in the right superior frontal and left inferior frontal regions that were more activated for number semantics in dyscalculics relative to controls. No significant relationship was observed (respectively: *r* = 0.22, F(1,39) = 2.0, *p =* 0.1, *ns*; and *r* = 0.26, F(1,39) = 2.7, *p =* 0.1, *ns*) and we found no evidence that faster dyscalculics had more or less parietal grey matter than slower dyscalculics [t(9) = 0.32, *p* = 0.76, *ns*].

## 4 Discussion

This study investigated how adults with developmental dyscalculia were able to make accurate although inefficient semantic judgements on numbers. To achieve this, a series of novel features were introduced in our study, which included: (1) an experimental paradigm with the same semantic tasks based on numerical and object names stimuli which allowed us to identify effects specific to number semantic processing; (2) a baseline task with the same stimuli as the number semantic tasks and matched for response requirements but not involving number semantic processing; (3) an analysis looking at the areas supporting residual processes in adults with dyscalculia, in addition to areas under-activated in dyscalculia relative to controls; (4) a further analysis looking at the structural integrity of parietal lobes in dyscalculia relative to controls; and (5) regressing out the possible impact of RT-related processes on any number-parietal activation. Based on these features, our behavioural results showed that accuracy in participants with dyscalculia did not differ from controls when judging the quantity or other semantic features of numbers; likewise, the two groups did not differ in the equivalent tasks with object names. However, dyscalculics' response times were significantly slower than controls only in the number semantic tasks. To account for this accurate but inefficient processing of numbers, we looked at brain activation in which participants with dyscalculia and control groups differed or did not differ. Common activation for dyscalculics and controls was observed in several parietal regions including the bilateral IPS, the right supramarginal gyrus, as well as the right inferior frontal regions. In addition, dyscalculics activated the right superior and the left inferior frontal gyrus significantly more than controls. This over-activation was detected only in fast but not slow adults with dyscalculia. This suggests that when dyscalculics activated these areas sufficiently, their response times in the in-scanner tasks were within the normal range. This link between BOLD response and number performance is unlikely to be uniquely driven by stimulus- or response-related factors that typically correlate with response times as there was no correlation with performance in other tasks that also measured RTs. Moreover, these frontal over-activations could not account for RTs per se as they were present in fast dyscalculics but not in equally fast controls.

Overall our results point to the following issues. First, they reinforce the idea that in some adults with dyscalculia there are residual number skills; second, they provide novel evidence for the brain systems supporting these residual number skills; third, they highlight a difference between accurate and efficient number performance in dyscalculics; fourth, they emphasize heterogeneity within the population with dyscalculia; and fifth, they indicate the important role of the right superior and left inferior frontal regions in maintaining dyscalculics' number performance. Residual number performance has been observed in some previous studies (Censabella and Noël, 2005; Kaufmann et al., 2009; Kucian et al., 2006; Landerl et al., 2004; Rotzer et al., 2008; Rubinsten and Henik, 2005) but with no account of the neuronal mechanisms supporting it as we have done in this investigation. Likewise, a difference between accurate and efficient performance has not been emphasized in previous studies of people with dyscalculia either because only some indexes of performance have been reported (e.g. accuracy but no RTs, Iuculano et al., 2008; Kucian et al., 2011), or because the focus of the study was different even when a discrepancy between accuracy and speed of performance was present (e.g. Censabella and Noël, 2005; Price et al., 2007; Rubinsten and Henik, 2005). Here we showed that participants with dyscalculia continued to make accurate numerical judgements, consistent with some previous reports (e.g. Censabella and Noël, 2005; Kaufmann et al., 2009; Kucian et al., 2006; Landerl et al., 2004; Rotzer et al., 2008; Rubinsten and Henik, 2005). The novelty of our results is to show that this accurate numerical performance was supported by activation in the parietal and inferior frontal regions which overall did not differ from control participants. These regions are within a network of areas that are typically engaged in number tasks (e.g. Cohen Kadosh et al., 2008; Dehaene et al., 2003) and that we previously observed in control participants performing the same tasks used in this study (Cappelletti et al., 2010). Moreover, by controlling the effect of RTs on number-parietal activations we showed that these activations were unlikely to be driven by other non-numerical factors such as response selection, as previously shown in controls (Cappelletti et al., 2010). The common activations found in adults with dyscalculia and in controls in the right IPS may seem in contrast with results of some previous studies claiming right parietal under-functioning in dyscalculia. However, the structural abnormalities we report are located in a similar parietal region as previously reported to be under-functioning in dyscalculia (MNI coordinates x = 33 y = − 50 z = 52; e.g. Kaufmann et al., 2011; Price et al., 2007). This therefore confirms the importance of this parietal area for number processing. Our novel evidence is to show that besides these right superior parietal abnormalities, other sub-regions of the parietal lobe and specifically the bilateral IPS (MNI coordinates: x = 32 y = − 72 z = 32 and x = − 34 y = − 58 z = 52) were contributing to maintaining dyscalculics' accurate number performance.

With respect to abnormally high activation in the frontal regions, we note that these are part of a larger network that has been previously associated with numerical processing in primates (Nieder and Dehaene, 2009). Moreover, the right superior frontal gyrus has sometimes been reported to be more activated in children with dyscalculia relative to numerically-normal children (Mussolin et al., 2010a; review by Kaufmann et al., 2011 reporting MNI co-ordinates: x = 0 y = 32 z = 44 which are similar to ours: x = 2 y = 30 z = 42), suggesting that people with dyscalculia may use it to compensate inefficient parietal activation.

The superior frontal gyrus is also thought to contribute to working memory processing and specifically to the mental manipulation and monitoring of information (Owen et al., 1996; Petrides et al., 1993). It is possible that high dyscalculic performers relied on more complex strategies to perform number tasks; for instance, they may have decomposed the numbers when comparing their magnitude (e.g. Moeller et al., 2009), or tried to visualize numbers along a line when categorizing them into dates or working/sleeping hours. Although some of these strategies are often used by numerically-normal controls (e.g. Moeller et al., 2009), dyscalculics may be less fluent when manipulating numbers, requiring extra working-memory resources to maintain numerical information available throughout the tasks, resulting in over-activated right superior frontal gyrus. These extra working memory resources may also be due to dyscalculics' reduced working memory in the context of numerical tasks (e.g. Fias et al., 2013). The right superior frontal lobe is also known for being involved in task switching, i.e. in reconfiguring the cognitive demands of a specific task once these demands have changed (Crone et al., 2006; Cutini et al., 2008). Our in-scanner experimental paradigm required such reconfiguration when switching from one task to another, for instance from number quantity (larger number among two) to number categorical task (working hour among two). Although task instructions were displayed before and during the task to facilitate remembering the task demands and task switching, it is possible that this switching was particularly demanding in participants with dyscalculia whose ability to navigate among number rules may not be as flexible as in numerically-normal participants.

Dyscalculics' over-activation of the left inferior frontal gyrus could also been explained in terms of the increased demand of some of the cognitive functions sub-served by this area. For instance, the left inferior frontal gyrus has been associated with response inhibition (Swick et al., 2008), with the resolution of interference or conflict that may occur when a strongly activated representation must be suppressed (Nelson et al., 2009; Thompson-Schill, 2005). Our number tasks required evoking different semantic representations, for instance based on a quantity (e.g. larger/smaller number) or on a categorical (e.g. sleeping/working time) criterion with no correspondence with each other, such that for instance the larger number did not necessarily coincide with the later time. Participants were therefore required to suppress the ‘quantity’ criterion when performing the number categorical task, and the ‘categorical’ criterion when performing the quantity tasks. This operation, which numerically-normal participants performed effortlessly, may have required additional resources in adults with dyscalculia, resulting in over-activation of the left inferior frontal area.

Dyscalculics' activation in the right superior and left inferior frontal lobes and in the posterior IPS correlated with response times in the number semantic tasks such that the higher the activation the faster the response, with a significant difference between slow and fast dyscalculic performers. Although these effects were detected at a group level, analyses of the individual performance indicated that they were significant only in efficient dyscalculic performers. The distinction between slow and fast adults with dyscalculia highlights the heterogeneity of numerical abilities within this population. At a theoretical level, heterogeneity in dyscalculia has been hypothesized to be relatively common (e.g. Landerl and Goebel, 2013; Rubinsten and Henik, 2009), although this referred to the fact that dyscalculia often is co-morbid with other disorders such as dyslexia, working memory or attentional deficits, at least in developing populations (Butterworth and Kovas, 2013). Instead, here heterogeneity refers to the variability within a sample of adult participants with dyscalculia with no additional behavioural disorders (Dowker, 2005). By exploring the impact of this variability on brain activity, we could identify brain regions associated with accurate but inefficient number processing in some of our dyscalculics, a novel approach that has so far only been used in numerically-normal subjects (e.g. Grabner et al., 2007).

## 5 Conclusions

We investigated structural abnormalities and under-functioning systems in adults with dyscalculia together with the systems supporting their residual number processing. We showed that despite reduced grey-matter volume in some right parietal regions, accurate semantic judgment on numbers was maintained by activations common with controls in parietal and frontal areas in addition to activations stronger than controls in right superior and left inferior frontal areas. Activation in these regions may reflect working memory, task-switching or inhibition processes required by high dyscalculic performers to achieve in the number semantic tasks. The difference between slow and fast dyscalculic performers in the right IPS and in the frontal regions highlights the importance of considering heterogeneity within dyscalculia.

## Competing financial interests

The authors declare no competing financial interests.

## Figures and Tables

**Table 1:** Performance in IQ subtasks in participants with dyscalculia. Percentile and standard deviation in brackets.

Tasks performed	All DD (N = 11)	Individual DD										
		1	2	3	4	5	6	7	8	9	10	11
IQ^a^	112.3 (15.1)	104	150	94	98	106	114	113	103	120	113	120
Verbal scale	109.5 (11.5)	104	na	91	91	119	112	121	106	116	112	123
Vocabulary^b^	71.7 (23.1)	84	na	50	**25**	95	63	63	63	84	95	95
Similarities^b^	70.1 (22.8)	84	na	**25**	37	75	63	99	75	84	84	75
Arithmetic^b^	**19.7 (19.8)**	37	**5**	50	**9**	50	**1**	**16**	**1**	37	**9**	**2**
Digit span^b^	63.5 (24.7)	**25**	50	75	37	99	75	84	37	75	50	91
Performance scale	105.1 (9.6)	103	na	96	106	89	116	106	99	121	113	102
Block design^b^	66.8 (24.7)	63	na	84	63	**9**	91	75	50	95	63	75
Matrices^b^	72.4 (17.8)	50	na	50	63	91	63	99	75	63	95	75

Legend: Impaired and borderline performance corresponding to 1 or 2 standard deviations below the 50th percentile is shown in bold and italics respectively. na = not available.

a WAIS-R (Wechsler, 1986). Full IQ calculated disregarding performance in the arithmetic sub-task.

b Percentile.

**Table 2:** Performance in the number tasks in participants with dyscalculia. Response times, percentile, percent correct or Weber fraction.

A. Tasks performed	Controls	All DD (N = 11)	Individual DD										
			1 (F)^a^	2 (F)^a^	3 (F)^a^	4 (F)^a^	5 (F)^a^	6 (F)^a^	7 (S)^a^	8 (S)^a^	9 (S)^a^	10 (S)^a^	11 (S)^a^
Dyscalculia screener^b^													
Capacity sub-scale		**2.9 (1.2)**	4.5	**2.5**	**1.5**	**2.5**	**2.5**	3.5	**2**	**2**	**2.5**	4.5	**1.5**
Dot — number matching			**3**	**3**	**1**	**3**	**3**	**3**	**3**	**2**	4	**3**	**2**
Number Stroop			6	**2**	**2**	**2**	**2**	4	**1**	**2**	**1**	6	**1**
Achievement sub-scale		**2.4 (0.9)**	**3**	3.5	**2**	**1.5**	**1.5**	**2**	**3**	**2.5**	3.5	**3**	**2**
Addition			4	**3**	**2**	**2**	**2**	**2**	4	**3**	4	**3**	**2**
Multiplication			**2**	4	**2**	**1**	**1**	**2**	**2**	**2**	**3**	**3**	**2**
GDA^c^	25–75	**23.5** (SD 18.7)	50	*25*	**9**	**1**	**16**	**16**	50	**1**	50	**16**	*25*
Number Acuity *wf*^d^	0.27 (SD 0.04)	**0.42** (SD 0.14)	*0.31*	**0.35**	**0.64**	*0.33*	**0.38**	*0.32*	**0.39**	**0.73**	**0.36**	**0.36**	**0.42**
Number comparison accuracy	95.6% (1.1)	95.2% (1.5)	98.5	97.05	100	94.1	98	98.5	100	100	97.05	100	100
RTs	602 ms (SD 109)	F^a^: **915**, S^a^:**1287**	*774*	**1009**	820	**1034**	**890**	**963**	**1183**	**1358**	**1406**	**1237**	**1249**
de^e^	95 ms (SD 32)	F^a^: **152**, S^a^: **252**	**302**	100	**160**	*49*	**206**	97	**208**	**396**	72	**160**	**425**
Number semantic^f^	1661 ms (SD 153)	F^a^:**1918,**S^a^:**2673**	**1953**	*1904*	*1897*	**2283**	**2300**	1169	**2346**	**3253**	**2432**	**2507**	**2828**

Legend: Except for the Dyscalculia Screener, impaired performance corresponded to 2 standard deviations below the controls mean performance or to 2 standard deviations below the 50th percentile, and it is shown in bold; borderline performance, corresponding to 1 standard deviation below controls mean performance or 1 standard deviations below the 50th percentile, is shown in italics. DD's performance was compared to controls in independent t-tests, and individual DD's performance was analysed with Crawford and Garthwaite (2002)*t*-test.

a F = fast, S = slow DD performers on the basis of performance in the in-scanner number semantic tasks.

b Stanine score ranging from 1 to 9 where ≤ 3 indicate an impairment (see Butterworth, 2003).

c Graded Difficulty Arithmetic Test, Jackson and Warrington, 1986; percentile score.

d Index of performance in a number discrimination task expressed as Weber fraction (*wf*, Halberda et al., 2008) which is sensitive to dyscalculia (e.g. Mazzocco et al., 2011; Piazza et al., 2010). SD = standard deviation is in brackets. DD's performance was compared to participants of Control Group 3.

e de = distance effect calculated from 68 trials corresponds to the difference in msec in responding to pairs of stimuli numerically close (1 vs 2) relative to far apart (1 vs 9). DD's performance was compared to participants of Control Group 3.

f Inside scanner performance (RTs in msec). Controls were 8 numerically-normal participants age-matched to the DD that took part in this study (Control Group 1, see text).

**Table 3:** Neuronal systems activated in adults with dyscalculia and in controls. In-scanner number semantic relative to number colour or to word semantic tasks in (A) common to participants with dyscalculia and controls, and (B) in participants with dyscalculia more than controls.

Area	H	Co-ordinates			Z scores			
					Nsem > Ncol^a^		Nsem > Wsem^b^	
		x	y	z	All	No. of voxels	All	No. of voxels
A.								
IPS	L	-34	− 58	52	**6.4**	128	3.5	16
	R	32	− 72	32	**6.9**	291	**5.4**	220
		30	− 58	50	**6.5**		**3.9**	90
SMG		38	− 44	40	**5.8**		*ns*	
IFG/IFS		48	10	30	**6.9**	91	4.0	62
		40	6	28	**6.5**		3.2	

					DD > controls	No. of voxels	DD > controls	No. of voxels

B.								
SFG	R	2	30	46	**5.0**	198	**5.5**	74
IFS	L	− 36	8	30	**4.9**	185	3.2	2

Legend: H = Hemisphere, L = Left; R = Right. Nsem = number semantics, Ncol = Number colour decision, Wsem = word semantics, Wcol = word colour decision. IPS = intra-parietal sulcus. SMG = Supramarginal gyrus. IFG/IFS = Inferior frontal gyrus/inferior frontal sulcus. SFG = superior frontal gyrus.

a Inclusively masked with Nsem > NCol in controls and Nsem > NCol in DD (*p* < 0.05).

b Inclusively masked with Nsem > Wsem in controls and Nsem > Wsem in DD (*p* < 0.05).

**Table 4:** Changes in brain activity and grey-matter volume in participants with dyscalculia relative to controls. Deviation in dyscalculics' activation or grey-matter density is expressed in standard deviations relative to controls' average.

Changes in brain activity and grey-matter volume	All DD (N = 11)	Individual DD										
		1 (F)^a^	2 (F)^a^	3 (F)^a^	4 (F)^a^	5 (F)^a^	6 (F)^a^	7 (S)^a^	8 (S)^a^	9 (S)^a^	10 (S)^a^	11 (S)^a^
Over-activation^b^												
• Right superior frontal	+ **2.5** (SD = 4.8)	**+ 2**	*+ 1*	**+ 2**	*+ 1*	**+ 2**	**+ 2**	*+ 1*	ns	ns	ns	*+ 1*
• Left inferior frontal	+ **4.3** (SD = 2.6)	**+ 2**	*+ 1*	**+ 2**	ns	**+ 2**	**+ 2**	*+ 1*	ns	*+ 1*	ns	*+ 1*
GM-volume decrease^b^:												
• Right parietal (36 -49 58)	**− 1.5** (SD = 0.05)	**− 2**	*− 1*	**− 2**	ns	**− 2**	**− 2**	ns	**− 2**	**− 2**	*− 1*	**− 2**

Legend: GM = grey-matter. SD = Standard deviation.

a F = fast, S = slow DD on the basis of performance in the in-scanner number semantic tasks.

b DDs' functional and structural abnormalities relative to controls are shown in bold, borderline performance in italics. Differences are measured in standard deviations relative to controls' activation or grey-matter density: ± 1 = borderline; ± 2 = impaired; ns = not significantly different from controls. Individual DD's performance was analysed with Crawford and Garthwaite (2002)*t*-test.
