# Supplementary material for: Residual number processing in dyscalculia
Source: Neuroimage Clin. 2013 Oct 14;4:18–28. doi: 10.1016/j.nicl.2013.10.004 (PMC3836281; doi:10.1016/j.nicl.2013.10.004)
Supplement: Supplementary file 1 — Appendix A. [file mmc1.docx]

**APPENDIX A - Residual number processing in dyscalculia**

**Tasks used to diagnose dyscalculia**

The **Dyscalculia Screener** is a standardized software that comprises four computer-controlled, item-timed tasks. The four tasks are divided into two subscales: a ‘capacity’ and an ‘achievement’ subscale, involving 2 tasks each (a dot-number matching and a number comparison task for the first subscale, and two math verification tasks for the second subscale; Butterworth, 2003, 2005). In each task, participants are required to make speeded responses by pressing one of two pre-defined keyboard keys. The software diagnoses dyscalculia on the basis of norms which look at performance expressed as an inverse efficiency score (median reaction times over accuracy, Butterworth, 2003, 2005; Landerl *et al*., 2004).

The **Graded Difficulty Arithmetical Test** is a standardized test based on twelve 2 to 3-digit addition and twelve 2 to 3-digit subtraction problems of progressive difficulty (e.g. from ‘13 + 15’ to ‘243+149’), orally presented one at a time for an oral answer which scored 1 point if correctly produced within 10 seconds (GDA, Jackson and Warrington, 1986). The WAIS-R arithmetical subtest (Wechsler, 1986) consists of a series of 20 arithmetical problems embedded in a text and orally presented for an oral answer. Correct answers produced within a maximum time (spanning from 15 to 60 seconds depending on the problem) were assigned 1 point.

**Number comparison** is a key test to assess intact number processing. This requires participants to indicate as fast as possible the larger of two Arabic numbers presented to the left and right of a central fixation point (‘F’ key with left index finger for greater numerical values on the left, ‘J’ key with right index finger for greater numerical value on the right). Thirty-six pairs of single-digit Arabic numbers (1 to 9) were individually presented. Stimulus pairs were centred along the horizontal line of the computer screen and displayed for 500msec each to the left or the right of the fixation cross which preceded each trial for 100ms; number stimuli were replaced by a black screen for a maximum of 4 seconds during which participants made an answer. After this, the following trial started immediately. Using a design similar to previous studies (e.g. Cappelletti et al. under review-b), the following numerical distances were used: 1 (e.g. 7 vs 6 or 5 vs 4; 8 trials), 2 (e.g. 3 vs 1 or 7 vs 9; 8 trials), 3 (e.g. 5 vs 8 or 4 vs 1; 8 trials), 4 (e.g. 2 vs 6 or 3 vs 7; 8 trials), 5 (e.g. 4 vs 9 or 1 vs 6; 4 trials), with an equal number of trials where the smaller digit was on the left or on the right within each numerical distance.

**Numerosity discrimination** was measured with a parametrically-modulated task requiring participants to indicate the more numerous set of stimuli (dots) among two simultaneously presented sets. The same experimental design, procedure and data analysis of a previous paradigm (Halberda et al., 2008) that has previously been used in participants with dyscalculia (Mazzocco et al., 2011; Piazza et al., 2010) was employed here. For each participant, we calculated the Weber fraction, an index of accuracy sensitive to dyscalculia following exactly the approach used in previous studies (Mazzocco et al., 2011; Piazza et al., 2010).

**SUPPLEMENTARY REFERENCES**

Butterworth, B. (2003). Dyscalculia Screener. London: Nelson Publishing Company Ltd.

Butterworth, B. (2005). Developmental dyscalculia. In J.I.D. Campbell (Ed)., *Handbook of mathematical Cognition* (pp. 455–467). Hove: Psychology Press.

Cappelletti, M., Chamberlain, R., Freeman, E.D., Kanai, R., Butterworth, B., Price, CJ., & Rees G. Dissociating numerical from continuous quantity skills. *Under review-b*.

Halberda J, Mazzocco MMM, Feigenson L. 2008. Individual differences in nonverbal number acuity predict maths achievement. Nature 455: 665-668.

Jackson, M., & Warrington, E.K. (1986). Arithmetic skills in patients with unilateral cerebral lesions. *Cortex. 22*, 611-620.

Landerl, K., Bevan, A., & Butterworth, B. (2004). Developmental dyscalculia and basic numerical capacities: a study of 8–9-year-old students. *Cognition, 93*, 99–125.

Mazzocco MMM, Feigenson L, Halberda J. 2011. Impaired acuity of the approximate number system underlies mathematical learning disability. Child Development. 82: 1224-1237.

Piazza M, Facoetti A, Trussardi AN, Berteletti I, Conte S, Lucangeli D, Dehaene S, Zorzi M. 2010. Developmental trajectory of number acuity reveals a severe impairment in developmental dyscalculia. Cognition. 116: 33–41.

Wechsler, D. (1986). *Wechsler Adult Intelligence Scale-Revised*. The Psychological Corporation.
